# TOX as a potential target for immunotherapy in lymphocytic malignancies

**DOI:** 10.1186/s40364-021-00275-y

**Published:** 2021-03-20

**Authors:** Chaofeng Liang, Shuxin Huang, Yujie Zhao, Shaohua Chen, Yangqiu Li

**Affiliations:** 1 Key Laboratory for Regenerative Medicine of Ministry of Education; Institute of Hematology, School of Medicine, Jinan University, Guangzhou, 510632 China; 2 Department of Anatomy and Molecular Embryology, Institute of Anatomy, Ruhr-University Bochum, 44801 Bochum, Germany

**Keywords:** Thymocyte selection-associated HMG BOX, Biological function, T cell exhaustion, Lymphocytic malignancies, Immune biomarker, Immunotherapy

## Abstract

TOX (thymocyte selection-associated HMG BOX) is a member of a family of transcriptional factors that contain the highly conserved high mobility group box (HMG-box) region. Increasing studies have shown that TOX is involved in maintaining tumors and promoting T cell exhaustion. In this review, we summarized the biological functions of TOX and its contribution as related to lymphocytic malignancies. We also discussed the potential role of TOX as an immune biomarker and target in immunotherapy for hematological malignancies.

## Background

TOX (thymocyte selection-associated HMG BOX), a transcription factor, belongs to the high mobility group box (HMG-box) superfamily. In 2002, Wilkinson et al. demonstrated for the first time that TOX plays an important role in the differentiation of CD4 + CD8+ double-positive thymocytes by gene microarray technology. Numerous studies have shown that TOX plays a vital role in the development and formation of various immune organs and cells such as CD4+ T cells and natural killer cells (NK cells). Recently, it has been reported that TOX may be involved in promoting CD8+ T cell exhaustion and the development of various malignant tumors [1, 2].

### Structure and biological function of TOX

**Fig. 1:**
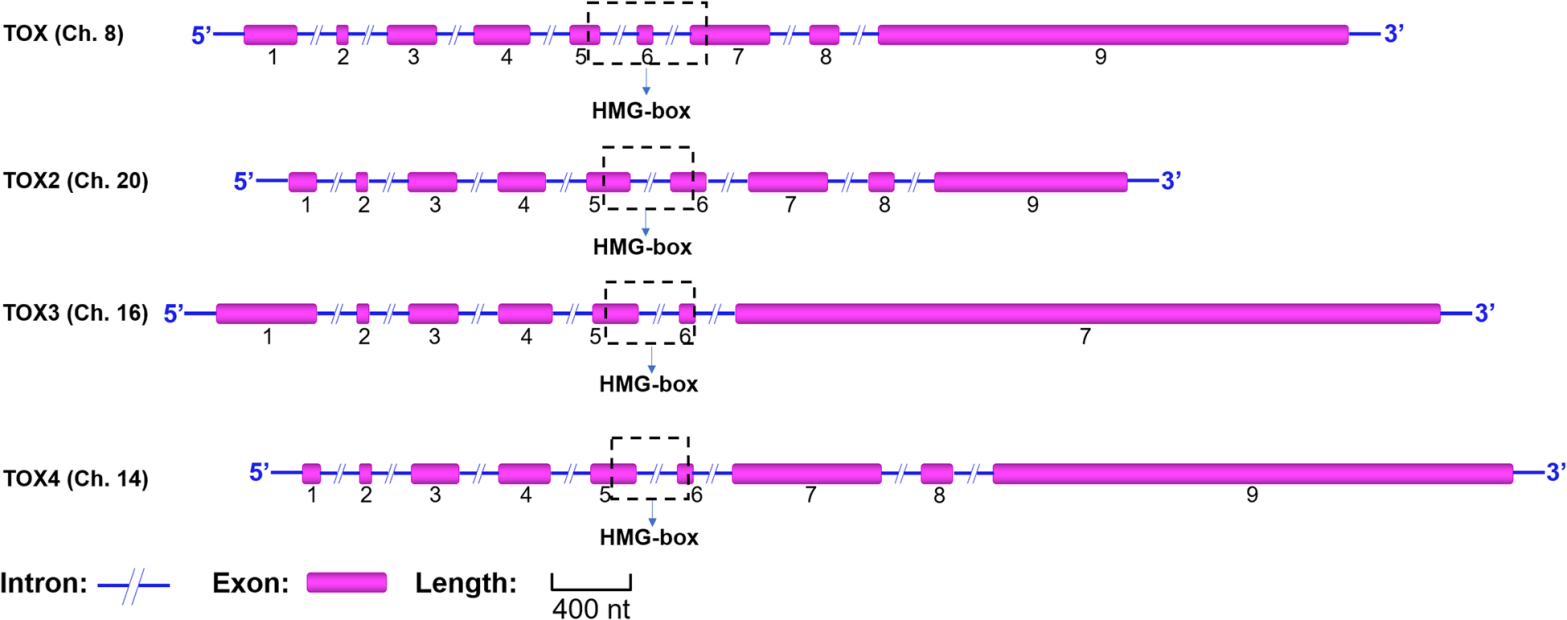
Schematic diagram of the *TOX* gene structure and location. The bars represent exons and the lines represent introns. The dotted box indicates the regions of exons that encode the HMG box. All exon lengths are drawn to scale with the scale bar indicating 400 nucleotides

There are four human *TOX* genes: *TOX* (also known as *TOX1*), *TOX2*, *TOX3,* and *TOX4*. These genes are located on different chromosomes and have different functions (Fig. 1, Table 1) [16].

**Table 1:** Biological function and the roles of TOX in cancer

Gene	Biological roles	Roles in cancer	References
*TOX* (8q12.1)	Immune system development	Formation of CD8+ T cell exhaustion Inhibited the expression of tumor suppressor genes Inhibited DNA repair	[3–6]
*TOX2* (20q13.12)	Altering chromatin Promoting T_FH_ cells and NK cell development	Formation of CD8+ T cell exhaustion	[4, 7, 8]
*TOX3* (16q12.1)	Protective factor for neurons	Highly expressed in breast cancers with poor prognosis Highly expressed and serve a tumor-suppressive role in lung cancer	[9–13]
*TOX4* (14q11.2)	Regulation of cell cycle and fate		[14, 15]

*TOX* Thymocyte selection-associated HMG BOX, *T*_*FH*_ *cells* T follicular helper cells, *NK cells* Natural killer cells

#### TOX

*TOX*, also known as KIAA0808, is located on q12.1 of human chromosome 8 and has one transcript. The length of the *TOX* mRNA is 4076 bp, and it contains nine exons that translate into a protein consisting of 526 amino acids. TOX is expressed in many human tissues, including hematopoietic and immune tissues, such as the tonsils, thymus, spleen, and bone marrow, and non-hematopoietic tissues, such as the intestines, lung, kidney, ovary, pancreas, breast and testis [17, 18]. In addition, TOX expression is relatively different in different tissues. For example, TOX is highly expressed in the thymus but has low expression in the spleen [19]. Overall, TOX plays a vital role in the immune system [3]. TOX expression gradually increases as NK cells begin to differentiate and decreases as NK cells mature. After silencing *TOX* by RNA interference, it was found that decreased *TOX* expression leads to a reduction in T-bet, thereby inhibiting the differentiation of NK cells [20, 21]. TOX is related to the formation of lymph nodes and Peyer’s patches, and knockout of *TOX* in mice resulted in the loss of lymph nodes and Peyer’s patches [21]. It has been found that both TOX and TOX2 are related to CD8+ T cell exhaustion [4]. Recently, novel findings showed that higher *TOX* expression is associated with better prognosis in breast cancer. Thus, it is considered that the alteration of *TOX* may be a potential prognostic marker for breast cancer [18].

#### TOX2

*TOX2*, also known as C20orf100, is located on q13.12 of human chromosome 20 and has four transcripts. The *TOX2* transcript encoding the longest isoform contains nine exons with an mRNA length of 2491 bp that is translated into a protein consisting of 506 amino acids. There are few studies on TOX2 at present. TOX2, similar to TOX, affects the development of NK cells by regulating the expression of T-bet. Interestingly, contrary to TOX, TOX2 is highly expressed in mature NK cells but not in the early stages of developing NK cells [7]. These data suggest that TOX and TOX2 may contribute to NK cell development at different time points.

#### TOX3

*TOX3*, also known as TNRC9, was initially found in the brain [22]. This gene is located on q12.1 of human chromosome 16 and has two transcripts. The *TOX3* transcript encoding the longest isoform contains seven exons, and the length of its mRNA is 4959 bp, which translated into a protein containing 576 amino acids. In 2009, Yuan et al. found that TOX3 is an important calcium-dependent transactivator in neurons and has a protective effect in neurons [9]. In patients with spinal cord injury, up-regulation of TOX3 can increase oligodendrocyte survival after injury, thereby reducing damage due to secondary degeneration during early spinal cord injury [10]. Genome-wide association studies (GWAS) have indicated that the variable expression of *TOX3* might play a different role in different tumors. For example, *TOX3* is highly expressed in breast cancer (particularly luminal breast cancer) and is associated with poor prognosis. Interestingly, this finding is opposite to the finding of *TOX* in breast cancer [18]. While in lung cancer, high expression of TOX3 is associated with better prognosis [11–13].

#### TOX4

*TOX4*, also known as KIAA0737, is located on q11.2 of human chromosome 14 and has two transcripts. The transcript encoding the longest isoform contains nine exons that leads to an mRNA 4514 bp in length that is translated into a protein containing 621 amino acids. TOX4 is involved in the regulation of the cell cycle and fate. In HEK293 cells, TOX4 forms a complex with protein phosphatase 1 (PP1), which participates in regulation of the cell cycle, proliferation, apoptosis, and other biological processes [23]. TOX4 reprograms somatic cells to form induced pluripotent stem cells (iPSCs) that differentiate these cells into a neuronal fate. Moreover, TOX4 is an indispensable factor for maintaining and establishing the pluripotency of embryonic stem cells and epiblast stem cells [14, 15].

### HMG-box of TOX

Although the number of exons in each TOX family member is different, the HMG-box region encoded by two or three exons in vertebrates is highly conserved among family members [24, 25]. The traditional HMG-box protein family can be divided into two classes. Class I HMG box group proteins are transcription factors containing a single HMG box that can bind DNA [25], and class II proteins have two DNA-binding motifs [26–28] that can bind DNA and alter chromatin structure [29–31]. TOX contains a single highly conserved HMG-box region and is a class I HMG-box protein. Most characteristics of TOX are similar to other HMG-box proteins; however, TOX lacks a key hydrophobic wedge residue that disables the induction of DNA bending [3]. In recent years, increasing studies have shown that TOX is a new class of HMG-Box proteins because TOX combines and recruits various proteins to form protein complexes that bind to DNA instead of directly binding DNA [5, 32]. Thus, it has been suggested TOX may function through a combination of different protein complexes on DNA to alter chromatin structure, which affects the expression of other genes.

### The role of TOX in T cell immunity

#### TOX and T cell differentiation

TOX was demonstrated to be involved in the proliferation and differentiation of T cells in *TOX* knock-out mice. The expression of TOX transiently increased during mouse β selection and positive selection in the thymus, and it decreased with the maturation of CD4+ and CD8+ T cells [16, 33, 34]. During the positive selection process, TOX determines the fate of CD8+ T cells by upregulating runt-related transcription factor 3 (RUNX3) and inhibiting the development of CD4+ T cells. Loss of TOX results in a considerable reduction in CD4+ T cells in mice, and CD4+ T cells cannot fully mature, thus losing some of their function [33]. TOX is associated with different types of cells in the thymus and is highly expressed in CD4 + CD8+ double-positive thymocytes but not in CD4-CD8- double-negative cells. Therefore, based on the different types of T cells that form in the thymus, T cells can be divided as TOX-dependent T cells (NKT cells, regulatory T cells), including CD4 + CD8+ double-positive thymus cells, and TOX-independent T cells (γδ T cells), including CD4-CD8- double-negative thymocytes [34]. TOX2 can alter chromatin and affect its function, promoting T follicular helper (T_FH_) cell development. Binding of the transcription factor B-cell lymphoma 6 (Bcl-6) to the *TOX2* locus fosters TOX2 expression, and the binding of TOX2 with DNA can alter the structure of chromatin to express relevant genes (such as *Bcl-6*, *Cxcr5*, and *Pdcd1*) and pathways (IL-6-STAT3, Notch, and Wnt). In *TOX2* knockout mice, the formation of T_FH_ cells decreased, impacting the development of B cells in the germinal center [8].

#### TOX is related to T cell senescence and exhaustion

**Fig. 2:**
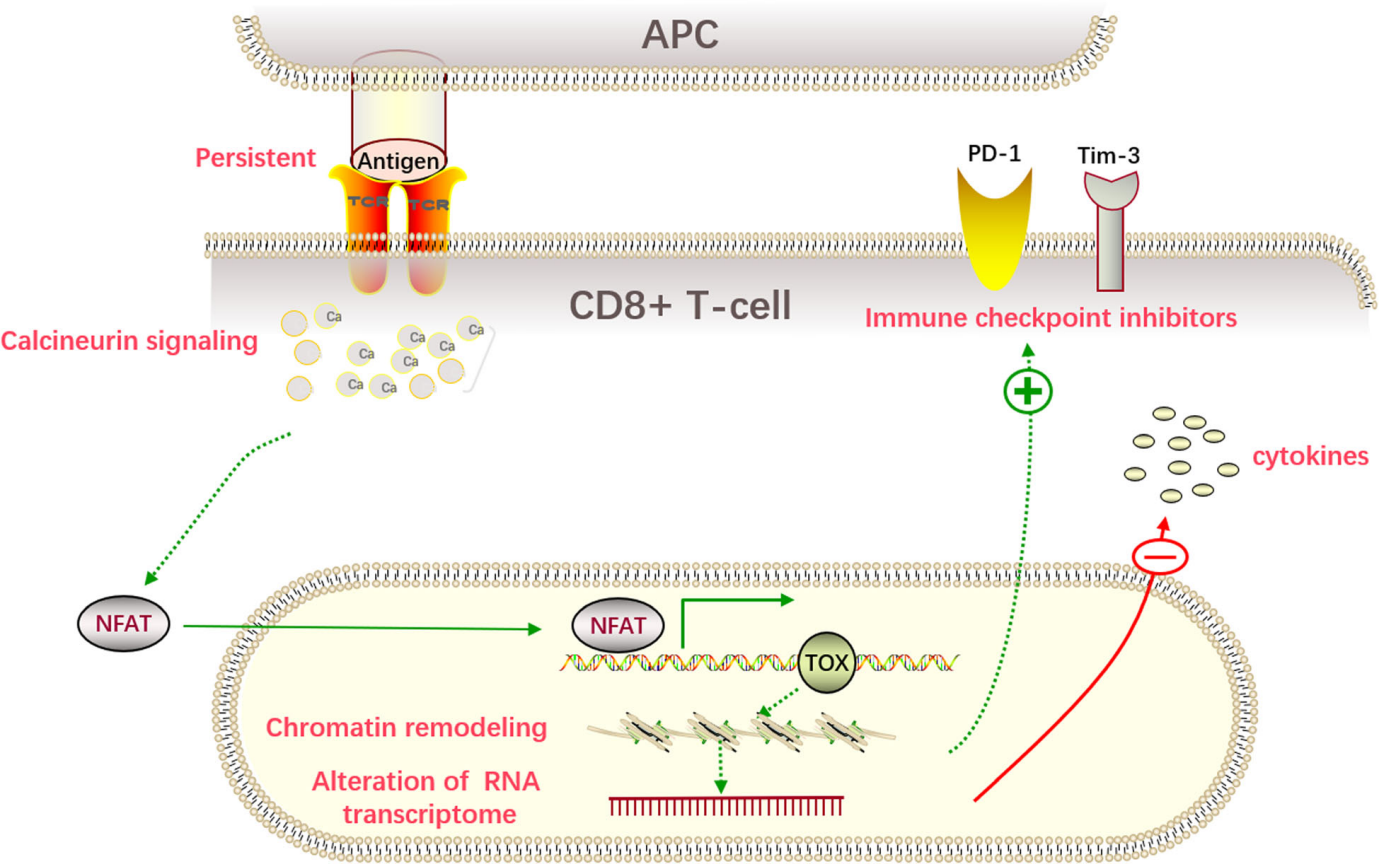
Schematic model of persistent antigen binding and continuous stimulation of CD8+ T cells. Persistent antigens activate calcium and NFAT signaling, inducing the expression of TOX in the nucleus. TOX remodels the chromatin and increases RNA transcription, which results in an increase in immune checkpoint inhibitor proteins and a decrease in cytokines. APC: antigen presenting cell, TCR: T cell receptor

T cell exhaustion is a term used to describe T cells under chronic antigen stimulation that alter or lose their effector function, and this is related to abnormal expression of immune checkpoint proteins in T cells [35–37]. Recently, many studies have shown that TOX is a crucial transcription factor involved in exhaustion of CD8+ T cells [1, 2, 4, 32, 38–41]. For example, in tumor-infiltrating CD8+ T cells from human melanoma and non-small cell lung cancer (NSCLC) samples, TOX had increased expression in CD8+ T cells with high expression of programmed cell death protein 1 (PD-1) [42]. The mechanism by which TOX promotes CD8+ T cell exhaustion may be similar to when antigens continuously stimulate CD8+ T cells where they activate calcium and NFAT signaling, thereby inducing the expression of the TOX in the nucleus. Once the pathway has begun, sustained TOX expression leads to chromosome recombination and changes in RNA transcription, inhibiting its differentiation into effector T cells and entering a state of exhaustion. In the exhaustion stage, the expression of immune checkpoint proteins such as PD-1, T cell immunoglobulin mucin-domain-containing-3 (Tim-3), and T cell immunoreceptor with Ig and ITIM domains (TIGIT), and cytotoxic T lymphocyte-associated molecule-4 (CTLA-4) and transcription factors, such as Eomes and TCF1, is increased, while the secretion of cytokines is impeded (Fig. 2) [2, 4, 32, 38–42]. Our recent study also showed that TOX is highly co-expressed with PD-1, Tim-3, and CD244 in CD3+ T cells, particularly in CD3 + CD8+ cells in patients with B cell non-Hodgkin’s lymphoma (B-NHL). Furthermore, the proportions of both TOX+ and TOX + PD-1+ regulatory T cells (Tregs) are also significantly increased in B-NHL patients [43]. This finding may further confirm that TOX contributes to promoting T cell exhaustion in lymphocytic malignancies.

### Alteration of TOX in lymphocytic malignancies

**Fig. 3:**
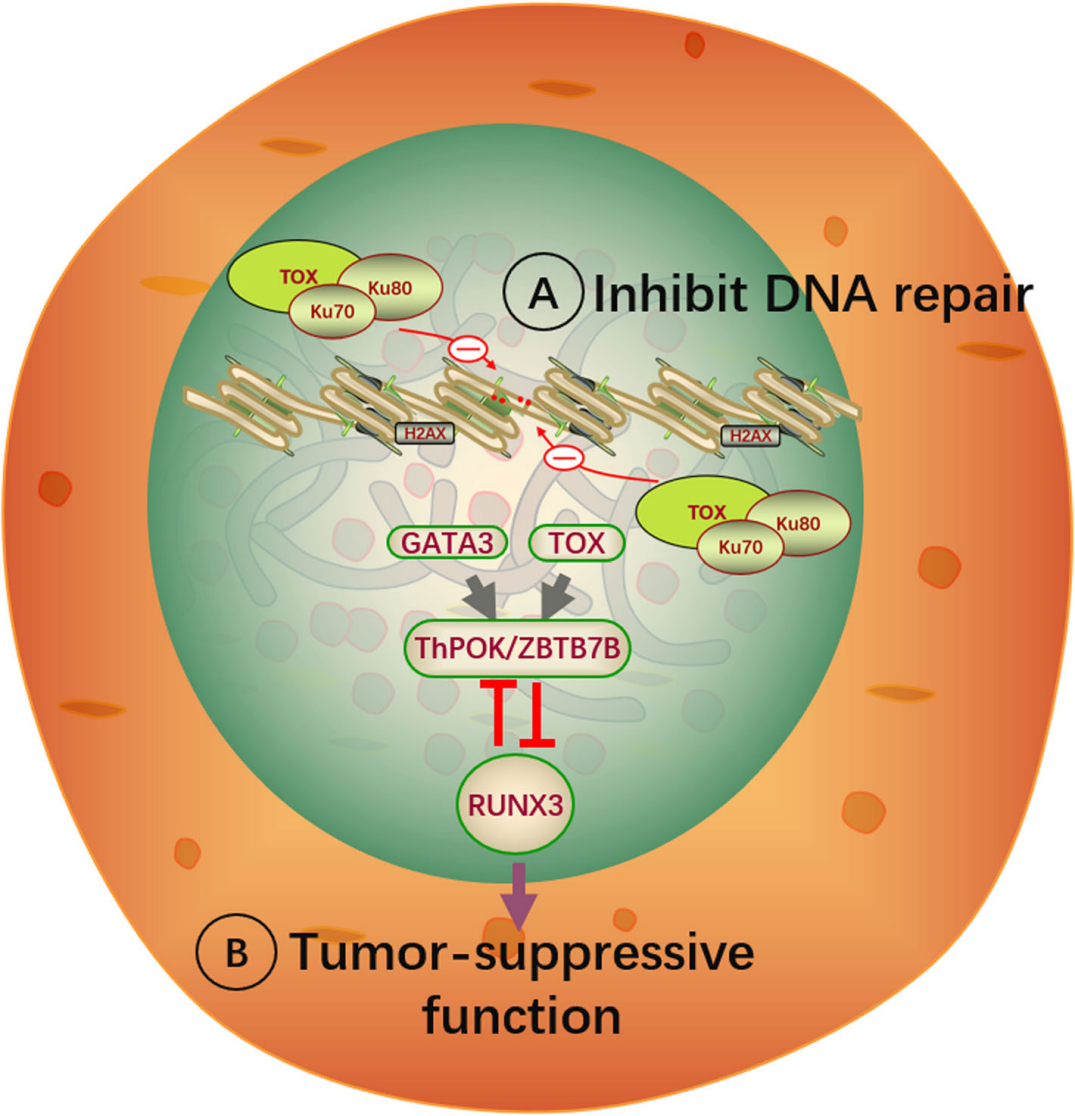
Mechanism of TOX function in T cell malignancies. **a** TOX binds to the repair factors KU70/KU80 and inhibits their function, causing abnormal NHEJ repair, leading to DNA repair inhibition. **b** TOX and GATA3 facilitate the downstream gene ThPOK to inhibit the expression of RUNX3, which leads to inhibition of its tumor-suppressive function

Genetic alterations in TOX have been characterized in lymphocytic malignancies, including acute lymphoblastic leukemia (ALL) and lymphoma. According to results from the Cancer Cell Line Encyclopedia database, compared with other cancers, TOX is highly expressed in ALL, particularly in T cell-ALL (T-ALL) [5]. The high expression of TOX is under regulation of the transcription factors T cell acute lymphoblastic leukemia protein 1 (TAL1) and LIM domain only 1/2 (LMO1/2) and binds to the repair factors KU70/KU80 to inhibit their function, causing abnormal non-homologous end joining (NHEJ) repair, thus inhibiting DNA repair in T-ALL cells and reducing genome stability. When *TOX* was knocked out in T-ALL cells, NHEJ repair could be restored (Fig. 3) [5]. It is well known that instability in the genome may be responsible for the development of T-ALL. TOX is even positively expressed in most ALL cases. In contrast, *TOX* deletion has also been reported in ALL patients. Molecular analysis of 205 cases with ALL demonstrated that approximately 4% of patients lacked *TOX* [44]. In addition, *TOX* deletion was found mostly during relapse for pediatric and adult ALL patients [45, 46]. Moreover, it was found in primary central nervous system lymphomas (PCNSL) that approximately 32% of patients have *TOX* deletion [47]. The role of *TOX* deletion in such patients remains unclear. Hence, it is worth investigating whether deletion is related to reduced T cell proliferation and anti-tumor function. Interestingly, the expression of TOX appears to be different in different types of lymphoma. In T cell lymphomas, TOX is highly expressed in mycosis fungoides (MF), precursor T lymphoblastic lymphoma, and angioimmunoblastic T-cell lymphoma (AITL), but it is expressed at a low level in peripheral T-cell lymphoma cell lymphoma (PTCL) and anaplastic large cell lymphoma (ALCL). In B-cell lymphoma, TOX is overexpressed in precursor B lymphoblastic lymphoma (B-LBL), diffuse large B cell lymphoma (DLBCL), follicular lymphoma (FL), and a small proportion of Burkitt lymphoma (BL) patients. There was no or only rare TOX expression in chronic lymphocytic leukemia (CLL), classical Hodgkin lymphoma, and myeloma [17].

### TOX inhibition for immunotherapy

TOX and TOX2 induced T cell exhaustion in different tumor mouse models and cancer patients as well as in patient hematological malignancies [4, 39–43]. TOX is associated with larger tumor size, lower differentiation, later TNM stage, and facilitating endocytic recycling of PD-1 [5]. Moreover, *TOX* knockdown in a mouse hepatocellular carcinoma (HCC) model or in tumor-infiltrating CD8+ T cells resulted in significant reduction in immune checkpoint proteins, including PD-1, CTLA-4, TIM-3, and TIGIT, ameliorating T cell exhaustion and promoting the antitumor effects of antigen-specific CD8+ T cells [32–34]. Moreover, in a chimeric antigen receptor-T cell (CAR-T) therapy tumor model, it was found that CAR-T cells doubly deficient for both TOX and TOX2 (*TOX* DKO) are more effective than either TOX-deficient or TOX2-deficient CAR-TILs (tumor-infiltrating lymphocytes) in suppressing tumor growth and prolonging the survival of tumor-bearing mice. *TOX* DKO CAR TILs demonstrated decreased expression of immune checkpoint proteins, increased cytokine secretion, and enhanced antitumor effects [3]. In contrast, knockdown of *TOX* in T-ALL cells by shRNAs exhibited disruption of the cell cycle and higher levels of apoptosis [5]. Up-regulating TOX may inhibit the expression of tumor suppressor genes, which may be a reason for tumor maintenance. It is known that reduction or deletion of the tumor suppressor gene RUNX3 occurs in many tumors [6, 48, 49]. The TOX-RUNX3 pathway inhibits the expression of RUNX3 when TOX is highly expressed, thereby affecting the tumor suppression function [50]. In cutaneous T-cell lymphoma, TOX and GATA3 can facilitate the expression of the downstream gene *ThPOK*, inhibiting the expression of RUNX3 (Fig. 3) [51]. Similarly, in Sézary syndrome (SS) cells, the expression of RUNX3 is reversed, and cell viability is reduced after TOX is suppressed by siRNA. Overall, targeted inhibition of TOX and TOX2 may include two therapeutic approaches for lymphocytic malignancies. First, directly inhibiting the proliferation of tumor cells as a targeted approach. Second, reversing T cell exhaustion to restore the anti-tumor function. Moreover, based on the finding that TOX expression was negatively correlated with the effects of PD-1 immunotherapy [40], it has been speculated that combined anti-TOX or anti-TOX2 and immune checkpoint inhibitor therapy may be a new approach for tumor immunotherapy.

## Conclusion and further directions

From the initial importance of TOX in the immune system for the development of various malignancies, the function of TOX and the possibility of its clinical application have been increasingly explored. In recent years, reversing T cell exhaustion has been the focus of immunotherapy. How to convert exhausted T cells into active T cells is a problem that many scientists are trying to solve. In hematological malignancies, CAR-T cell therapy has impressive therapeutic efficacy [52]; however, it also faces the drawback that CAR-T cells lose their effects due to rapid exhaustion after injection into patients [37, 52]. On this issue, TOX might be used as a specific target to reverse T cell exhaustion by reducing its expression activity, which can maintain the effects of CAR-T cells in patients for a longer period of time. Moreover, TOX has different characteristics in different lymphocytic malignancies, and the role of TOX in T cell exhaustion in patients with myeloid leukemia is worth further investigation [53]. Overall, TOX and TOX2 might be potential immune biomarkers and targets for hematological malignancy immunotherapy. Moreover, for patients with deleted TOX, it remains an open question how to design TOX-based targeted therapies.

## Data Availability

The material supporting the conclusions of this review are included within the article.

## Abbreviations

AILT: Angioimmunoblastic T-cell lymphoma
ALCL: Anaplastic large cell lymphoma
ALL: Acute lymphoblastic leukemia
Bcl-6: B-cell lymphoma 6
BL: Burkitt lymphoma
B-LBL: B lymphoblastic lymphoma
B-NHL: B cell non-Hodgkin’s lymphoma
CAR-T: Chimeric antigen receptor-T cell
CLL: Chronic lymphocytic leukemia
CTLA-4: Cytotoxic T lymphocyte-associated molecule-4
DLBCL: Diffuse large B cell lymphoma
FL: Follicular lymphoma
GWAS: Genome-wide association studies
HCC: Hepatocellular carcinoma
HMG-box: High mobility group box
iPSC: Induced pluripotent stem cells
LMO1/2: LIM domain only 1/2
MF: Mycosis fungoides
NHEJ: Non-homologous end joining
NK cells: Natural killer cells
NSCLC: Non-small cell lung cancer
PCNSL: Primary central nervous system lymphomas
PD-1: Programmed cell death protein 1
PP1: Protein phosphatase 1
PTCL: Peripheral T-cell lymphoma cell lymphoma
RUNX3: Runt-related transcription factor 3
SS: Sézary syndrome
TAL1: T cell acute lymphoblastic leukemia protein 1
T-ALL: T cell-ALL
T_FH_: T follicular helper cell
TILs: Tumor-infiltrating lymphocytes
Tim-3: T cell immunoglobulin mucin-domain-containing-3
TOX: Thymocyte selection-associated HMG BOX
Tregs: Regulatory T cells
